# *Rhinogobius immaculatus*, a new species of freshwater goby (Teleostei: Gobiidae) from the Qiantang River, China

**DOI:** 10.24272/j.issn.2095-8137.2018.065

**Published:** 2018-06-27

**Authors:** Fan Li, Shan Li, Jia-Kuan Chen

**Affiliations:** 1 Institute of Biodiversity Science, Ministry of Education Key Laboratory for Biodiversity Science and Ecological Engineering, Fudan University, Shanghai 200433, China; 2 Shanghai Ocean University, Shanghai 200090, China; 3 Shanghai Natural History Museum, Branch of Shanghai Science & Technology Museum, Shanghai 200041, China

**Keywords:** Gobiidae, *Rhinogobius*, New species, Qiantang River, China

## Abstract

A new freshwater goby, *Rhinogobius immaculatus* **sp. nov.**, is described here from the Qiantang River in China. It is distinguished from all congeners by the following combination of characters: second dorsal-fin rays I, 7–9; anal-fin rays I, 6–8; pectoral-fin rays 14–15; longitudinal scales 29–31; transverse scales 7–9; predorsal scales 2–5; vertebrae 27 (rarely 28); preopercular canal absent or with two pores; a red oblique stripe below eye in males; branchiostegal membrane mostly reddish-orange, with 3–6 irregular discrete or connected red blotches on posterior branchiostegal membrane and lower operculum in males; caudal-fin base with a median black spot; and no black blotch on anterior part of first dorsal fin in males.

## INTRODUCTION

The freshwater goby genus *Rhinogobius* Gill, 1859, is currently comprised of 74 valid species (Huang et al., 2016; Suzuki et al., 2017; Takahashi & Okazaki, 2017) widely distributed in East Asia, including Russia (Bogutskaya et al., 2008), Japan (Akihito et al., 2002), Korea (Regan, 1908b), China (Chen & Shao, 1996; Wu & Zhong, 2008), Philippines (Herre, 1927), Vietnam (Chen & Kottelat, 2005), Laos (Chen & Kottelat, 2003; Kottelat, 2001), Cambodia (Rainboth, 1996), and Thailand (Chen et al., 1999a). Most species of *Rhinogobius* from the islands of Japan and Taiwan are amphidromous (Chen & Shao, 1996; Lee & Chang, 1996; Sakai et al., 2000), whereas most species from eastern continental Asia and Hainan Island are non-diadromous (landlocked) (Chen et al., 1999a, 2002; Chen & Kottelat, 2005; Chen & Miller, 2014; Huang & Chen, 2007; Li & Zhong, 2009).

In total, 44 species of *Rhinogobius* have been recorded in China (Chen et al., 2008; Chen & Miller, 2014; Huang et al., 2016; Huang & Chen, 2007; Li et al., 2007; Li & Zhong, 2007, 2009; Wu & Zhong, 2008; Yang et al., 2008), eight of which have been reported from the Qiantang River basin originating in southeastern Anhui Province to eastern Zhejiang Province. These species include *R. aporus* (Zhong & Wu, 1998), *R. davidi* (Sauvage & de Thiersant, 1874), *R. cliffordpopei* (Nichols, 1925), *R. leavelli* (Herre, 1935a), *R. lentiginis* (Wu & Zheng, 1985), *R. niger* Huang, Chen & Shao, 2016, *R. similis* Gill, 1859, and *R. wuyiensis* Li & Zhong, 2007 (Chen et al., 1990; Li, 2011; Liu et al., 2011; Suzuki et al., 2016; Zheng, 1989; Zheng & Wu, 1985). Herein, we describe a new species from three tributaries of the Qiantang River, China.

## MATERIALS AND METHODS

Specimens for morphological examination were initially preserved in 6% formalin for seven days, and then transferred into 70% ethanol for permanent storage. Methods for morphometric measurements and meristic counts followed Nakabo (2002), with exceptions as indicated: Standard length (SL), head length, snout length, predorsal length, and preanal length were measured from the tip of the upper lip; Head depth and width were taken at the posterior margin of the preopercle; Body depth and width were taken at the origin of the anal fin. Vertebrae were counted from radiographs using the Kodak DXS 4000 system, and 3D reconstructed CT scans were made with the NSI-x50 system. Notations of cephalic sensory-canal pores and sensory-papillae rows followed Akihito et al. (2002) and Suzuki et al. (2017). Examined specimens in this study were deposited in the Biological Museum, Fudan University, Shanghai (FDU) and Shanghai Ocean University, Shanghai (SOU/SFU/SFC).

## RESULTS

*Rhinogobius immaculatus* **sp. nov.**

**Figure 1:**
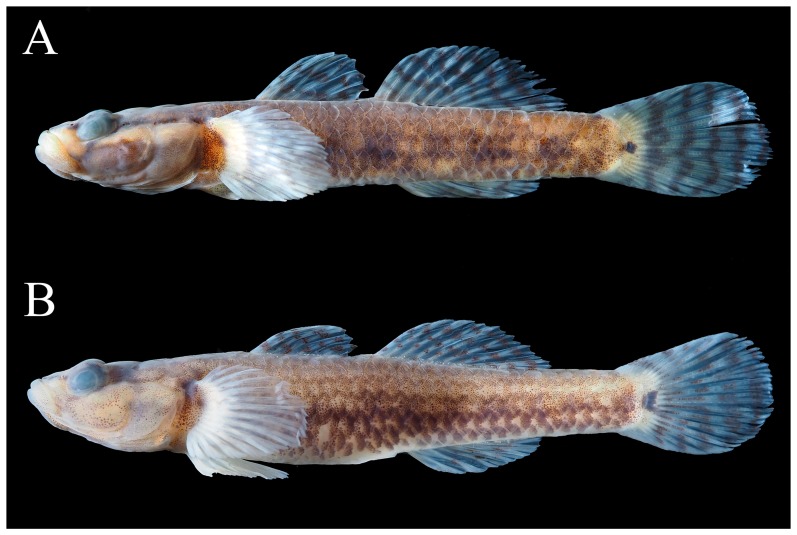
*Rhinogobius immaculatus* sp. nov.

**Figure 2:**
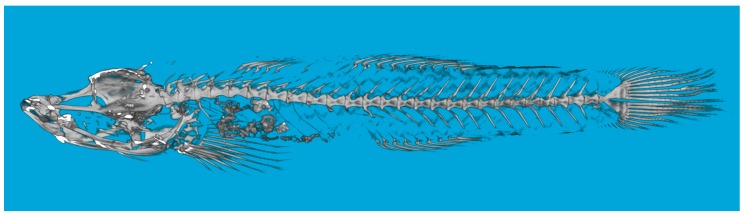
*Rhinogobius immaculatus* sp. nov.

**Figure 3:**
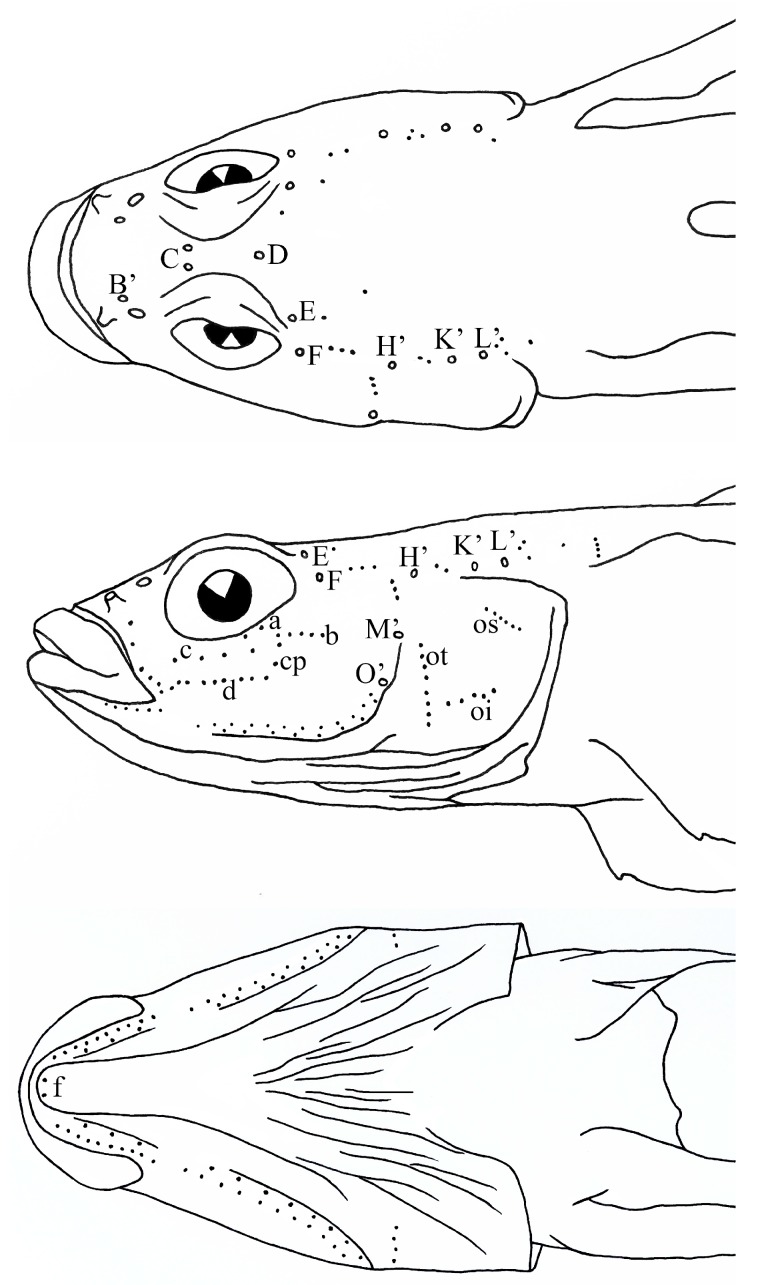
Cephalic lateral-line system of *Rhinogobius immaculatus* sp. nov.

**Figure 4:**
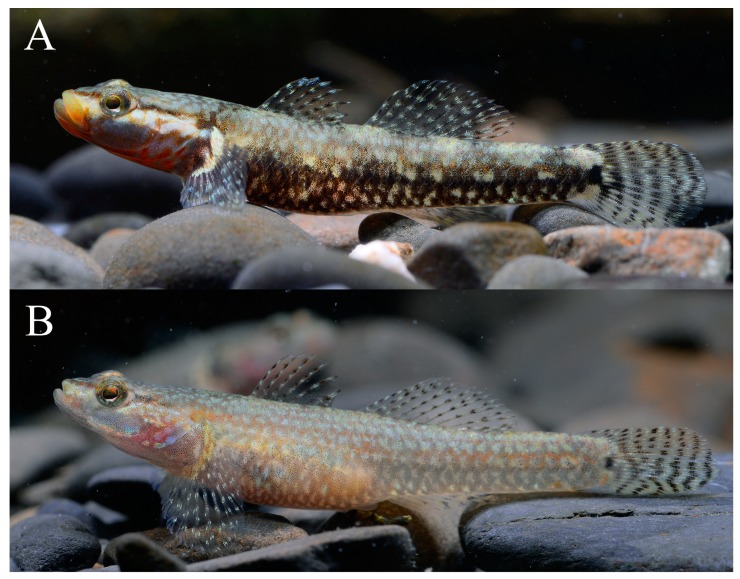
Male (A) and female (B) *Rhinogobius immaculatus* sp. nov.

**Figure 5:**
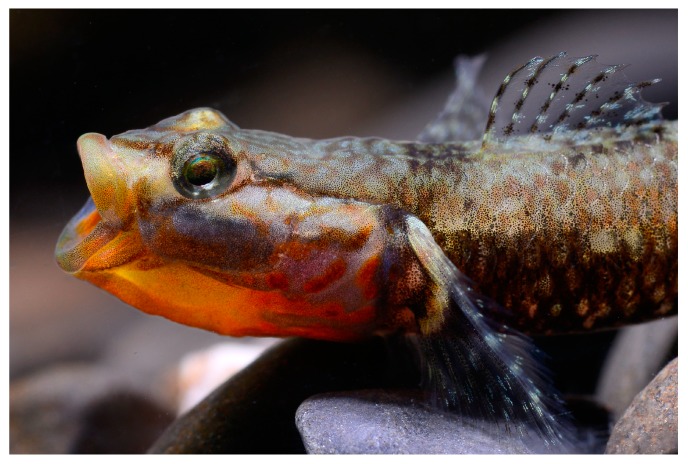
Coloration of head and first dorsal fin of male *Rhinogobius immaculatus* sp. nov.

Figure 1, Figure 2, Figure 3, Figure 4 and Figure 5; Table 1.

A: FDU 1010001, holotype, 25.7 mm SL, male; B: FDU 1107004, paratype, 24.4 mm SL, female.

FDU 1107006, paratype, 22.2 mm SL, female. 3D reconstructed CT scan (with concealment of left skull).

FDU 1107003, paratype, 20.5 mm SL.

Specimens collected from Xiuning County, Anhui Province, China. Specimens not preserved.

Specimen collected from Xiuning County, Anhui Province, China. Specimen not preserved.

**Table 1:** Meristic and morphometric data for holotype and paratypes of *Rhinogobius immaculatus* sp. nov.

	Holotype	Paratypes	
	Male	Males	Females
Number	1	5	18
Standard length (mm)	25.7	19.2–22.1	20.9–25.2
First dorsal-fin rays	VI	VI (5)	V (1); VI (15)
Second dorsal-fin rays	I,9	I,7 (2); I,8 (3)	I,7 (1); I,8 (15); I,9 (2)
Anal-fin rays	I,7	I,6 (2); I,7 (3)	I,6 (2); I,7 (14); I,8 (2)
Pectoral-fin rays	15	15 (5)	14 (7); 15 (11)
Longitudinal scales	30	29 (2); 30 (2); 31 (1)	29 (8); 30 (8); 31 (2)
Transverse scales	7	8 (5)	7 (6); 8 (11); 9(1)
Predorsal scales	4	2 (1); 3 (2); 4 (1); 5 (1)	2 (4); 3 (6); 4 (4); 5 (4)
Vertebrae	27	unknown	27 (9); 28 (1); unknown (8)
**Morphometry**			
% standard length			
Head length	27.8	28.1–29.7	25.0–27.7
Head depth	15.0	15.2–16.1	13.8–14.8
Head width	19.4	17.9–20.6	17.1–18.3
Body depth of anal-fin origin	14.1	14.2–15.1	14.1–16.3
Body width of anal-fin origin	11.2	12.3–13.3	10.7–12.5
Snout length	6.7	6.1–7.3	5.6–6.9
Lower jaw length	8.6	7.2–9.3	6.3–7.6
Orbit diameter	7.9	6.7–8.3	6.7–8.5
Predorsal length	36.7	35.5–38.1	35.0–36.3
Preanal length	61.1	58.4–59.7	58.7–61.9
Caudal peduncle length	25.0	24.8–27.1	24.7–28.6
Caudal peduncle depth	7.6	7.7–10.9	7.4–11.1
Depressed 1st dorsal-fin length	19.2	17.5–18.3	16.8–18.8
Depressed 2nd dorsal-fin length	32.7	27.0–31.0	26.5–29.7
Depressed anal-fin length	24.0	22.4–25.5	21.6–24.2
Pectoral-fin length	24.4	22.9–25.8	20.5–23.8
Pelvic-fin length	16.7	17.0–18.1	15.3–18.7
Caudal-fin length	24.7	23.0–26.5	21.6–25.3

Numbers in parentheses are numbers of specimens with a given count.

**Holotype**: FDU 1010001, male, 25.7 mm SL; a tributary of Qiantang River, Fuchunjiang Town, Tonglu County, Zhejiang Province, China; 7 October 2010. 

**Paratypes**: FDU 1010002, female, 26.3 mm SL; same data as holotype. FDU 0905001–0905002, 2 males, 19.2–19.7 mm SL; FDU 0905003–0905006, 4 females, 20.9–21.4 mm SL; same locality as holotype; 4 May 2009. FDU 1107001–1107003, 3 males, 20.5–22.1 mm SL; FDU 1107004–1107014, 11 females, 22.2–25.2 mm SL; a tributary of Qiantang River, Xikou Town, Xiuning County, Anhui Province, China; 23 July 2011. FDU 1710001–1710002, 2 females, 20.4–20.7 mm SL; a tributary of Qiantang River, Dongyangjiang Town, Dongyang City, Zhejiang Province, China; 3 October 2017. 

**Diagnosis**: Most similar to *Rhinogobius wuyanlingensis* in number of vertebrae (27) and preopercular canal pores (2 or 0 vs. 2), but differing by fewer pectoral-fin rays (14–15 vs. 17–18), fewer anal-fin rays (I, 6–7 vs. I, 8), fewer transverse scales (7–9, modally 8 vs. 9–10), absence of a black blotch on anterior part of first dorsal fin in males (vs. present), and branchiostegal membrane mostly reddish-orange, with irregular blotches posteriorly in males (vs. with red stripes). 

**Description**: The morphometric and meristic data of the holotype and paratypes are shown in Table 1. The following features can describe the new species: Body cylindrical anteriorly and compressed posteriorly. Head sub-cylindrical. Eye large, dorsolateral. Mouth oblique, with lower jaw tip anterior-most, jaw forming 45${}^{\circ }$ angel with body axis; corner of mouth reaching below anterior margin of orbit in adult males, not reaching below anterior margin in females. Vertebral counts 11+16=27 (10) or 11+17=28 (1) (Figure 2).

First dorsal-fin rays V–VI (modally VI); second dorsal-fin rays I, 7–9 (modally I, 8); anal-fin rays I, 6–8 (modally I, 7); pectoral-fin rays 14–15 (modally 15); pelvic-fin rays I, 5; segmented caudal-fin rays 9+8, including branched rays 7+7; dorsal procurrent rays 6–8, ventral procurrent rays 5–7. First dorsal fin with third or fourth spine longest, no filamentous spines; rear tip not reaching origin of second dorsal fin when depressed in both sexes. Second dorsal and anal fins short-based, tip of depressed rays far from dorsal and ventral origins of procurrent caudal-fin rays. Origin of anal fin inserted below base of third and fourth rays of second dorsal fin. Pectoral fin elliptical, central rays longest; rear extension far from vertical of anus when depressed. Pelvic fin disc rounded. Rear edge of caudal fin rounded.

Longitudinal scales 29–31 (28–30 on body, 0–1 on caudal fin); transverse scales 7–9 (modally 8); predorsal scales 2–5. Body with moderately ctenoid scales. Anterior predorsal area, head, pectoral base, and prepelvic area naked. Posterior predorsal area and belly with cycloid scales. Anterior-most predorsal scale not reaching vertical through upper end of gill opening.

Head pores present. Nasal extension of anterior oculoscapular canal with terminal pores B’ at vertical between anterior and posterior nostrils. Anterior interorbital section of oculoscapular canal separated, with paired pore C. Single pore D in posterior interorbital region. Postorbital region with paired pore E. Lateral section of anterior oculoscapular canal with anterior pore F and terminal pore H’. Posterior oculoscapular canal short, reduced (absent in 14 specimens), with two terminal pores K’ and L’. Gap between anterior and posterior oculoscapular canals larger than length of posterior oculoscapular canal. Preopercular canal short, reduced (absent in eight specimens), with two terminal pores M’ and O’. Sensory-papillae row a short, with two or three papillae below orbit. Row b short, about half length of orbit. Rows c and d longer, not extending to vertical line of rear margin of orbit. Single cp papilla. Row f paired (Figure 3). 

**Color in life**: Ground color light brown. Snout with pair of reddish brown stripes united at tip of snout. A reddish oblique stripe below eye in males, not reaching rear edge of mouth; obscure in females. Cheek and opercle with irregular reddish lines, branchiostegal membrane mostly reddish-orange, with 3–6 irregular discrete or connected red blotches on posterior branchiostegal membrane and lower operculum in males; absent in females. Flank with 6–7 irregular discrete or connected black blotches. First dorsal fin with 3–4 rows of interphased black and white spots. A black blotch on anterior part of first dorsal fin absent in both sexes. Second dorsal fin with 4–5 rows of interphased black and white spots. Pectoral fin proximally white, posterior part with 3–4 rows of interphased black and white spots. Pectoral-fin base with irregular blackish pigmentation, usually darker in upper part. Caudal fin with 5–6 rows of interphased black and white spots. Caudal-fin base with a median black blotch. Pelvic fin and anal fin with slight irregular black pigmentation (Figure 4A–B, Figure 5).

**Figure 6:**
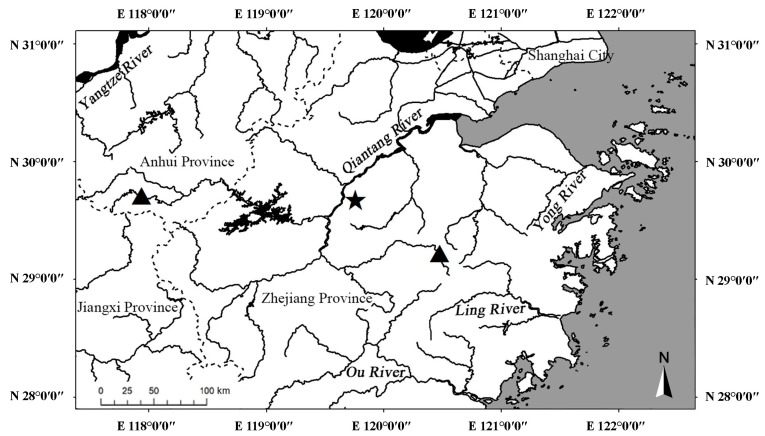
Sampling localities of *Rhinogobius immaculatus* sp. nov.

**Distribution and ecology**: Known only from streams of the Qiantang River basin in Zhejiang and Anhui Provinces, China (Figure 6). Most often found in shallow (10–50 cm deep) low-gradient streams, with sand and gravel mixed substrate.

★: type locality; ▴: other sampling localities.

Adult *Rhinogobius immaculatus* **sp. nov.** are small in size. The smallest female with mature oocytes was 22.4 mm SL. The largest specimen collected in the field was 26.3 mm SL. The largest captive specimen kept in an aquarium for 29 months was 32.8 mm SL. 

**Etymology**: The specific name, *immaculatus*, is derived from Latin *in* (without) and *maculatus* (spotted), an adjective, alluding to the absence of a black blotch on the anterior part of the first dorsal fin in adult males.

## DISCUSSION

Number of vertebrae is frequently used for species identification in the genus *Rhinogobius* (Chen et al., 2008; Huang et al., 2016; Lee & Chang, 1996; Suzuki et al., 2017; Takahashi & Okazaki, 2017; Yang et al., 2008). Among the current 74 valid species in *Rhinogobius*, 36 possess 27 or more vertebrae (Huang et al., 2016), as also found in the newly described species, and 28 possess less than 27 vertebrae (Suzuki et al., 2016, 2017; Takahashi & Okazaki, 2017). The vertebral number of the remaining species remains unknown.

In the genus *Rhinogobius*, only *R. wuyanlingensis* Yang, Wu & Chen, 2008 and *R. lindbergi* Berg, 1933 are known to have two preopercular canal pores (Huang et al., 2016; Sakai et al., 2000). *Rhinogobius immaculatus* **sp. nov.** closely resembles *R. wuyanlingensis* in number of vertebrae (27), preopercular canal pores (2 or 0 vs. 2), presence of predorsal scales, and small adult size, but differs from *R. wuyanlingensis* in fewer pectoral-fin rays (14–15 vs. 17–18), fewer anal-fin rays (I, 6–7 vs. I, 8), fewer transverse scales (7–9, modally 8 vs. 9–10), absence of a black blotch on the anterior part of the first dorsal fin in males (vs. present), and branchiostegal membrane mostly reddish-orange, with irregular blotches posteriorly in males (vs. with red stripes) (Yang et al., 2008). *Rhinogobius immaculatus* **sp. nov.** shares the same number of vertebrae (27) and preopercular canal pores (2 or 0 vs. 2) with *R. lindbergi*, but differs in fewer pectoral-fin rays (14–15 vs. 19–20), fewer anal-fin rays (I, 6–7 vs. I, 9), and more predorsal scales (2–5 vs. 0) (Berg, 1933; Sakai et al., 2000).

*Rhinogobius immaculatus* **sp. nov.** can be distinguished from the other 34 species with 27 or more vertebrae as follows: from *R. albimaculatus* Chen, Kottelat & Miller, 1999a, *R. boa* Chen & Kottelat, 2005, *R. changtinensis* Huang & Chen, 2007, *R. chiengmaiensis* Fowler, 1934, *R. duospilus* (Herre, 1935b), *R. filamentosus* (Wu, 1939), *R. flumineus* (Mizuno, 1960), *R. henryi* (Herre, 1938), *R. honghensis* Chen, Yang & Chen, 1999c, *R. lineatus* Chen, Kottelat & Miller, 1999a, *R. linshuiensis* Chen, Miller, Wu & Fang, 2002, *R. longyanensis* Chen, Cheng & Shao, 2008, *R. lungwoensis* Huang & Chen, 2007, *R. maculicervix* Chen & Kottelat, 2000, *R. mekongianus* (Pellegrin & Fang, 1940), *R. milleri* Chen & Kottelat, 2003, *R. nammaensis* Chen & Kottelat, 2003, *R. niger*, *R. ponkouensis* Huang & Chen, 2007, *R. sulcatus* Chen & Kottelat, 2005, *R. taenigena* Chen, Kottelat & Miller, 1999a, *R. vermiculatus* Chen & Kottelat, 2003, *R. wangchuangensis* Chen, Miller, Wu & Fang, 2002, *R. wangi* Chen & Fang, 2006, *R. xianshuiensis* Chen, Wu & Shao, 1999b, and *R. yaoshanensis* (Luo, 1989) by fewer preopercular canal pores (2 or 0 vs. 3) and absence of a black blotch on the anterior part of the first dorsal fin in males (vs. present) (Chen et al., 2008; Huang et al., 2016; Wu & Zhong, 2008); from *R. genanematus* Zhong & Tzeng, 1998, and *R. parvus* (Luo, 1989) by more predorsal scales (2–5 vs. 0–4, usually 0 in *R. parvus*, and 0 in *R. genanematus*) and fewer preopercular canal pores (2 or 0 vs. 3) (Chen et al., 2008); from *R. cheni* (Nichols, 1931) by fewer longitudinal scales (29–31 vs. 34) and fewer preopercular canal pores (2 or 0 vs. 3) (Chen et al., 2008); from *R. szechuanensis* (Tchang, 1939) by presence of oculoscapular canal (vs. absent) and more predorsal scales (2–5 vs. 0) (Chen et al., 2008; Wu & Zhong, 2008); from *R. davidi* and *R. multimaculatus* (Wu & Zheng, 1985) by more predorsal scales (2–5 vs. 0) and fewer vertebrae (27 vs. 28 in *R. davidi*, 29 in *R. multimaculatus*) (Chen & Miller, 1998; Yang et al., 2008; Zheng & Wu, 1985); from *R. rubromaculatus* Lee & Chang, 1996 by fewer transverse scales (7–9 vs. 10–13) and fewer predorsal scales (2–5 vs. 9–13) (Chen & Shao, 1996); and from *R. lentiginis* by fewer transverse scales (7–9 vs. 10–11), absence of spots on cheek (vs. present), and absence of a black blotch on the anterior part of the first dorsal fin in males (vs. present) (Li, 2011; Zheng & Wu, 1985).

In addition to differences in vertebral number, *Rhinogobius immaculatus* **sp. nov.** can be distinguished from the 28 species with less than 27 vertebrae as follows: from *R. aporus*, *R. changjiangensis* Chen, Miller, Wu & Fang, 2002, *R. leavelli*, *R. nandujiangensis* Chen, Miller, Wu & Fang, 2002, *R. reticulatus* Li, Zhong & Wu, 2007, *R. rubrolineatus* Chen & Miller, 2008, *R. sagittus* Chen & Miller, 2008, *R. sangenloensis* Chen & Miller, 2014, *R. variolatus* Chen & Kottelat, 2005, *R. virgigena* Chen & Kottelat, 2005, and *R. wuyiensis* by absence of a black blotch on the anterior part of the first dorsal fin in males (vs. present) (Herre, 1935a; Li & Zhong, 2007; Wu & Zhong, 2008; Zhong & Wu, 1998); from *R. biwaensis* Takahashi & Okazaki, 2017, *R. brunneus* (Temminck & Schlegel, 1845), *R. candidianus* (Regan, 1908a), *R. delicatus* Chen & Shao, 1996, *R. fluviatilis* Tanaka, 1925, *R. formosanus* Oshima, 1919, *R. gigas* Aonuma & Chen, 1996, *R. henchuenensis* Chen & Shao, 1996, *R. kurodai* (Tanaka, 1908), *R. lanyuensis* Chen, Miller & Fang, 1998, *R. maculafasciatus* Chen & Shao, 1996, *R. mizunoi* Suzuki, Shibukawa & Aizawa, 2017, *R. nagoyae* Jordan & Seale, 1906, *R. nantaiensis* Aonuma & Chen, 1996, and *R. ogasawaraensis* Suzuki, Chen & Senou, 2012 by fewer pectoral-fin rays (14–15 vs. more than 16) and presence of a median black blotch on the caudal fin base (vs. absent) (Akihito et al., 2002; Chen & Shao, 1996; Suzuki & Chen, 2011; van Oijen et al., 2011); from *R. similis* by fewer pectoral-fin rays (14–15 vs. 18–19) and fewer predorsal scales (2–5 vs. 8–12) (Gill, 1859; Suzuki et al., 2016); and from *R. zhoui* Li & Zhong, 2009 by fewer predorsal scales (2–5 vs. 10–12) and absence of white margin on each median fin (vs. present).

*Rhinogobius immaculatus* **sp. nov.** can be distinguished from the 10 species with unknown vertebral number as follows: from *R. bedfordi* (Regan, 1908b), *R. bucculentus* (Herre, 1927), *R. carpenteri* Seale, 1910, and *R. philippinus* (Herre, 1927) by fewer longitudinal scales (29–31 vs. more than 35); from *R. cliffordpopei* by more predorsal scales (2–5 vs. 0) (Nichols, 1925); from *R. fukushimai* Mori, 1934, and *R. imfasciocaudatus* Nguyen & Vo, 2005 by fewer pectoral-fin rays (14–15 vs. 18) (Nguyen, 2005); from *R. liui* Chen & Wu, 2008 by fewer pectoral-fin rays (14–15 vs. 19), fewer longitudinal scales (29–31 vs. 36–39), and more predorsal scales (2–5 vs. 0) (Wu & Zhong, 2008); from *R. shennongensis* (Yang & Xie, 1983) by fewer pectoral-fin rays (14–15 vs. 18–19) and fewer longitudinal scales (29–31 vs. 31–33); and from *R. sowerbyi* Ginsburg, 1917 by fewer longitudinal scales (29–31 vs. 35–36) and fewer anal-fin rays (I, 6–7 vs. I,8).

## COMPARATIVE MATERIAL

*Rhinogobius aporus*: FDU 200910103, topotypes, 14 specimens, 23.0–31.5 mm SL; Ou River, China: Zhejiang Province: Jinyun County; 9 October 2009. FDU 201010106, 16 specimens, 21.9–37.5 mm SL; Qiantang River, China: Zhejiang Province: Wuyi County; 9 October 2010. FDU 201710101, 23 specimens, 23.5–35.7 mm SL; Ou River, China: Zhejiang Province: Pan’an County; 4 October 2017.

*Rhinogobius cliffordpopei*: FDU 201010105, 6 specimens, 25.3–32.0 mm SL; Qiantang River, China: Zhejiang Province: Yongkang City; 8 October 2010. FDU 201309101, 11 specimens, 24.2–36.9 mm SL; Yangtze River, China: Hunan Province: Lianyuan City; 26 September 2013.

*Rhinogobius davidi*: FDU 200905101, 6 specimens, 22.5–27.0 mm SL; Qiantang River, China: Zhejiang Province: Tonglu County; 24 May 2009. FDU 201110101, 18 specimens, 18.4–37.5 mm SL; Qiantang River, China: Zhejiang Province: Kaihua County; 7 October 2011.

*Rhinogobius changtinensis*: FDU 201711101, topotypes, 18 specimens, 28.2–38.9 mm SL; Han River, China: Fujian Province: Changting County; 28 November 2017.

*Rhinogobius duospilus*: FDU 200711101, 24 specimens, 21.8–33.0 mm SL; China: Guangdong Province: Shenzhen City; November 2007. FDU 201310101, 6 specimens, 31.1–39.6 mm SL; Pearl River, China: Guangxi Province: Jinxiu County; 1 October 2013. FDU 201712101, 6 specimens, 22.2–28.7 mm SL; Pearl River, China: Guangxi Province: Nanning City; 26 December 2017.

*Rhinogobius formosanus*: FDU 201512101, 13 specimens, 29.8–54.2 mm SL; China: Fujian Province: Fuqing City; 12 December 2015.

*Rhinogobius genanematus*: FDU 200910102, topotypes, 11 specimens, 19.5–28.4 mm SL; Ling River, China: Zhejiang Province: Xianju County; 8 October 2009. FDU 201007102, 14 specimens, 22.6–32.3 mm SL; Ling River, China: Zhejiang Province: Tiantai County; 27 July 2010.

*Rhinogobius gigas*: FDU 201409101, 4 specimens, 40.8–62.4 mm SL; Hsiukuluan River, China: Taiwan: Taitung County; 20 September 2014.

*Rhinogobius honghensis*: FDU 201002101, 28 specimens, 35.8–49.4 mm SL; Red River, China: Guangxi Province: Napo County; February 2010.

*Rhinogobius leavelli*: FDU 201010104, 7 specimens, 34.5–44.2 mm SL; Qiantang River, China: Zhejiang Province: Tonglu County; 7 October 2010. FDU 201108101, 9 specimens, 23.1–51.3 mm SL; Lingshui River, China: Hainan Province: Baoting County; 21 August 2011.

*Rhinogobius lentiginis*: FDU 201007102, topotypes, 17 specimens, 25.4–40.3 mm SL; Ling River, China: Zhejiang Province: Tiantai County; 27 July 2010. FDU 200706101, 21 specimens, 24.8–37.2 mm SL; Cao’e River (tributary of Qiantang River), China: Zhejiang Province: Xinchang City; 10 June 2007.

*Rhinogobius lindbergi*: FDU 201208101, topotypes, 5 specimens, 19.5–25.1 mm SL; China: Heilongjiang Province: Harbin City; 28 August 2012.

*Rhinogobius linshuiensis*: FDU 201108101, topotypes, 13 specimens, 20.2–37.5 mm SL; Lingshui River, China: Hainan Province: Baoting County; 5 August 2011.

*Rhinogobius multimaculatus*: FDU 201107101, topotypes, 5 specimen, 19.7–35.2 mm SL; Tiaoxi River China: Zhejiang Province: Anji County; 24 July 2011.

*Rhinogobius niger*: FDU 200910102, topotypes, 3 specimens, 29.9–34.2 mm SL; Ling River, China: Zhejiang Province: Xianju County; 8 October 2009. FDU 201007101, 15 specimens, 22.1–43.9 mm SL; Yong River, China: Zhejiang Province: Fenghua City; 26 July 2010. FDU 201007102, 10 specimens, 17.5–33.7 mm SL; Cao’e River (tributary of Qiantang River), China: Zhejiang Province: Xinchang City; 27 July 2010.

*Rhinogobius reticulatus*: SFU 07001, holotype, male, 33.6 mm SL; Min River, China: Fujian Province: Fuzhou City; August 2005. SFU 07002, paratype, female, 30.4 mm SL; SFU 07003–07006, paratypes, 4 males, 27.3–35.8 mm SL; SFU 07007–07011, paratypes, 6 females, 25.6–34.5 mm SL; same data as holotype. FDU 201010102, 14 specimens, 20.5–27.8 mm SL; Min River, China: Fujian Province: Pucheng County; 5 October 2010.

*Rhinogobius rubromaculatus*: FDU 201503101, 8 specimens, 19.5–34.8 mm SL; Shihwen River, China: Taiwan: Pingtung County; 29 March 2015.

*Rhinogobius shennongensis*: FDU 201404101, topotypes, 5 specimens, 46.5–50.3 mm SL; Yangtze River, China: Hubei Province: Shennongjia Forestry District; 23 April 2014.

*Rhinogobius similis*: FDU 201009101, 6 specimens, 44.1–62.5 mm SL; Yangtze River, China: Jiangxi Province: Wuyuan County; 3 Sep. 2010. FDU 201010101, 5 specimens, 39.5–52.4 mm SL; Qiantang River, China: Zhejiang Province: Changshan; County; 3 September 2010. FDU 201708101, 2 specimens, 36.5–36.9 mm SL; Perfume River, Vietnam: Hue City; 29 August 2017.

*Rhinogobius szechuanensis*: FDU 201005101, topotypes, 7 specimens, 41.6–58.9 mm SL; Yangtze River, China: Sichuan Province: Qionglai City; May 2010.

*Rhinogobius yaoshanensis*: FDU 201310101, topotypes, 6 specimens, 23.6–41.5 mm SL; Pearl River, China: Guangxi Province: Jinxiu County; 1 October 2013. FDU 201310102, 3 specimens, 39.6–43.9 mm SL; Pearl River, China: Guangxi Province: Bama County; 7 October 2013.

*Rhinogobius wuyanlingensis*: FDU 200710101, 8 specimens, 176–27.7 mm SL; Ao River, China: Zhejiang Province: Pingyang City; 12 October 2007. FDU 200910101, 19 specimens, 20.3–27.8 mm SL; Dajing River, China: Zhejiang Province: Yueqing City; 4 October 2009.

*Rhinogobius wuyiensis*: SFU 07101, holotype, male, 39.2 mm SL; Qiantang River, China: Zhejiang Province: Wuyi County; July 2006. SFU 07102, paratype, female, 37.0 mm SL; SFU 07103–07109, paratypes, 7 males, 32.0–41.6 mm SL; SFU 07110–07118, paratypes, 8 females, 31.6–38.6 mm SL; same data as holotype. FDU 201010103, 8 specimens, 19.9–27.5 mm SL; Qiantang River, China: Zhejiang Province: Lanxi City; 6 October 2010. FDU 201007101, 12 specimens, 17.6–35.9 mm SL; Yong River, China: Zhejiang Province: Fenghua City; 26 July 2010.

*Rhinogobius xianshuiensis*: SFC 2257–2258, paratypes, 2 specimens, 24.8–28.8 mm SL; Xianshui River, China: Fujian Province: Xianyou County; 19 August 1994. FDU 201407101, topotypes, 9 specimens, 23.1–32.0 mm SL; Xianshui River, China: Fujian Province: Xianyou County; 16 July 2014.

*Rhinogobius zhoui*: SOU 0804001, holotype, male, 33.4 mm SL; Huang River, China: Guangdong Province: Lianhua County; April 2008. SOU 0804002, paratype, female, 31.7 mm SL; SOU 0804003–0804008, paratypes, 6 males, 26.6–36.1 mm SL; SOU 0804009–0804013, paratypes, 5 females, 30.3–32.1 mm SL; same data as holotype.
